# Hyperpolarized Carbon-13 MRI for Early Response Assessment of Neoadjuvant Chemotherapy in Breast Cancer Patients

**DOI:** 10.1158/0008-5472.CAN-21-1499

**Published:** 2021-10-08

**Authors:** Ramona Woitek, Mary A. McLean, Stephan Ursprung, Oscar M. Rueda, Raquel Manzano Garcia, Matthew J. Locke, Lucian Beer, Gabrielle Baxter, Leonardo Rundo, Elena Provenzano, Joshua Kaggie, Andrew Patterson, Amy Frary, Johanna Field-Rayner, Vasiliki Papalouka, Justine Kane, Arnold J.V. Benjamin, Andrew B. Gill, Andrew N. Priest, David Y. Lewis, Roslin Russell, Ashley Grimmer, Brian White, Beth Latimer-Bowman, Ilse Patterson, Amy Schiller, Bruno Carmo, Rhys Slough, Titus Lanz, James Wason, Rolf F. Schulte, Suet-Feung Chin, Martin J. Graves, Fiona J. Gilbert, Jean E. Abraham, Carlos Caldas, Kevin M. Brindle, Evis Sala, Ferdia A. Gallagher

**Affiliations:** 1 Cancer Research UK Cambridge Centre, University of Cambridge, Cambridge, United Kingdom.; 2 Department of Radiology, University of Cambridge, Cambridge, United Kingdom.; 3 Department of Radiology, Addenbrooke's Hospital, Cambridge University Hospitals NHS Foundation Trust, Cambridge, United Kingdom.; 4 Department of Biomedical Imaging and Image-guided Therapy, Medical University of Vienna, Vienna, Austria.; 5 Cancer Research UK Cambridge Institute, University of Cambridge, Li Ka Shing Center, Cambridge, United Kingdom.; 6 MRC Biostatistics Unit, University of Cambridge, Cambridge, United Kingdom.; 7 Department of Oncology, Cambridge Breast Cancer Research Unit, Addenbrooke's Hospital, Cambridge University Hospitals NHS Foundation Trust, Cambridge, United Kingdom.; 8 Department of Oncology, Addenbrooke's Hospital, Cambridge University Hospitals NHS Foundation Trust, Cambridge, England.; 9 Molecular Imaging Laboratory Cancer Research UK Beatson Institute, Glasgow, United Kingdom.; 10 Institute of Cancer Sciences, University of Glasgow, Glasgow, United Kingdom.; 11 RAPID Biomedical, Rimpar, Germany.; 12 Population Health Sciences Institute, Newcastle University, Newcastle-upon-Tyne, United Kingdom.; 13 GE Healthcare, Munich, Germany.; 14 Department of Biochemistry, University of Cambridge, Cambridge, United Kingdom.

## Abstract

Hyperpolarized ^13^C-MRI is an emerging tool for probing tissue metabolism by measuring ^13^C-label exchange between intravenously injected hyperpolarized [1–^13^C]pyruvate and endogenous tissue lactate. Here, we demonstrate that hyperpolarized ^13^C-MRI can be used to detect early response to neoadjuvant therapy in breast cancer. Seven patients underwent multiparametric ^1^H-MRI and hyperpolarized ^13^C-MRI before and 7–11 days after commencing treatment. An increase in the lactate-to-pyruvate ratio of approximately 20% identified three patients who, following 5–6 cycles of treatment, showed pathological complete response. This ratio correlated with gene expression of the pyruvate transporter *MCT1* and lactate dehydrogenase A (*LDHA*), the enzyme catalyzing label exchange between pyruvate and lactate. Analysis of approximately 2,000 breast tumors showed that overexpression of *LDHA* and the hypoxia marker *CAIX* was associated with reduced relapse-free and overall survival. Hyperpolarized ^13^C-MRI represents a promising method for monitoring very early treatment response in breast cancer and has demonstrated prognostic potential.

**Significance:**

Hyperpolarized carbon-13 MRI allows response assessment in patients with breast cancer after 7–11 days of neoadjuvant chemotherapy and outperformed state-of-the-art and research quantitative proton MRI techniques.

## Graphical Abstract

**Figure:**
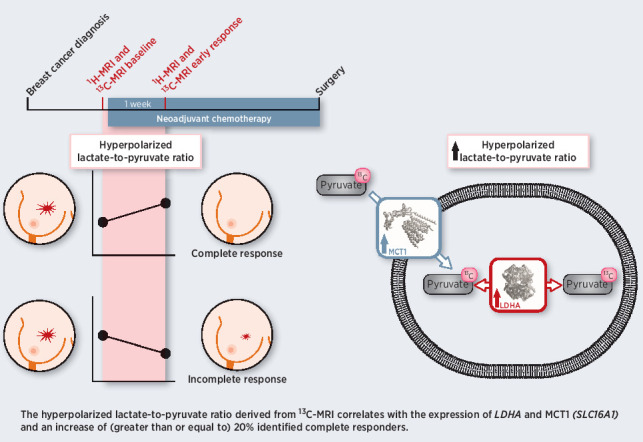

## Introduction

Neoadjuvant chemotherapy (NACT) is the standard-of-care treatment for 17%–40% of patients with operable early-stage breast cancer, particularly patients with HER2-positive (HER2^+^) and triple-negative breast cancer (TNBC; ref. 1). NACT can be used in downstaging of locally advanced breast cancer when breast conservation is considered and for testing novel drugs. Pathological complete response (pCR) at surgery indicates a favorable prognosis and rates of pCR have recently been shown to reach 68%–80% in patients receiving carboplatin or dual HER2 blockade (2). The response to NACT can guide decisions regarding additional adjuvant systemic therapy in nonresponders (3, 4) or de-escalation of therapy if an early response is identified (5).

Early prediction of pCR on imaging can be used to decrease side effects from non-efficacious drugs in nonresponders and could allow these patients to receive alternative regimens or investigational agents. Although early response assessment in breast cancer can be undertaken with multiparametric proton MRI (^1^H-MRI) using dynamic contrast-enhanced MRI (DCE-MRI) and diffusion-weighted imaging (DWI), a recent meta-analysis demonstrated substantial heterogeneity in sensitivity and specificity between studies (6, 7).

Aggressive breast cancers, such as HER2^+^ and TNBC, often switch to glycolysis, either as a result of aerobic glycolysis (the Warburg effect) or due to hypoxia, leading to increased intratumoral lactate production (8–10). This switch can be detected by intravenous injection of hyperpolarized [1–^13^C]pyruvate and monitoring the subsequent exchange of the hyperpolarized ^13^C-label between pyruvate and lactate using ^13^C MRI. This experimental clinical imaging tool has been explored in a number of cancer types (11–16). We have demonstrated the feasibility of using hyperpolarized ^13^C-MRI to assess patients with different subtypes of breast cancer, where higher levels of lactate labeling were observed in higher grade tumors, including TNBCs (11). In the majority of preclinical studies to date, successful treatment response was demonstrated by a decrease in tumor lactate labeling, which can be observed as early as 24 hours after cytotoxic treatment in a range of cancer models, including breast cancer (17). However, in a small number of animal studies the opposite effect occurred, with an increase in ^13^C-lactate signal following a successful response to therapy (18, 19). We have also recently described the first clinical example of response assessment in human breast cancer following NACT using hyperpolarized ^13^C-MRI, showing a decreased flux of hyperpolarized ^13^C-label into lactate after one complete NACT cycle of 3 weeks. DCE-MRI in this patient incorrectly predicted a poor response to therapy (16). Important questions remain about the temporal changes in lactate labeling and how widely applicable this response is across patients and tumor types.

Most studies define early response in breast cancer as that observed after a full cycle or several weeks of NACT (6). We have previously used a similar time point for the first proof-of-principle study in which hyperpolarized ^13^C-MRI was used to monitor treatment response in breast cancer (16). However, there is an unmet need for very early response assessment in patients with breast cancer, ideally within days or a week of treatment, to allow patients to rapidly change to the most appropriate treatment. Hyperpolarized ^13^C-MRI is a promising candidate technique for this very early response assessment as metabolic changes in response to treatment have been shown to occur on this timescale (17, 20–22). This prospective clinical study was designed to assess the potential added value of hyperpolarized ^13^C-MRI for very early response assessment in patients with aggressive breast cancer (TNBC or HER2^+^) undergoing neoadjuvant treatment in comparison with advanced multiparametric proton MRI techniques. The aim was to determine whether responders could be identified by an early change in lactate ^13^C-labeling. We have also explored the relationship between lactate labeling in individual patients and the expression, at an RNA level, of those genes that may influence pyruvate metabolism, demonstrating the prognostic significance of this pattern of gene expression in a large group of patients with breast cancer.

## Patients and Methods

### Patient recruitment

Local research ethics committee approval was obtained for this prospective study [National Research Ethics Service Committee East of England, Cambridge South, Research Ethics Committee number 15/EE/0378; National Institute for Health Research (NIHR) portfolio number 30388]. Written informed consent was obtained from seven patients diagnosed with invasive carcinoma of the breast between 2018 and 2020. Data for the baseline exam of one of these patients were included in a previous publication (11).

pCR was defined as the absence of residual invasive carcinoma in the resected breast specimen at surgery, regardless of the presence of DCIS.

### MRI

Multinuclear breast MRI, including ^13^C-MRI with hyperpolarized [1–^13^C]pyruvate was performed at baseline before treatment initiation (median number of days between baseline ^13^C-MRI and treatment initiation was 4). For one of the baseline scans, the ^1^H-MRI was performed four days before the ^13^C-MRI for technical reasons. Early follow-up scans for response assessment, including ^13^C-MRI were performed 7–11 days after the first dose of NACT was administered (median = 7 days).

### ^1^H-MRI and postprocessing

All patients underwent proton breast MRI on a clinical 3 T scanner (MR750, GE Healthcare). The inbuilt ^1^H body coil was used to acquire three-dimensional fast gradient echo scout images and subsequently T_1_-weighted axial and coronal fast spoiled gradient echo images were used to plan the subsequent ^13^C-MRI (for specifications see Supplementary Materials). After completion of ^13^C-MRI, diagnostic quality proton breast MRI was undertaken in the prone position using a dedicated eight-channel phased array receive-only breast coil as described previously (11, 23). Details regarding acquisition, reconstruction, and analysis of DCE-MRI and diffusion weighted MRI are provided in the Supplementary Materials.

### ^13^C-MRI and postprocessing

Details regarding pharmacy kit and pyruvate sample preparation and hyperpolarization are provided in the Supplementary Materials.

In five patients, spectral–spatial ^13^C-imaging was performed (patients 2, 4, 5, 6, and 7 in Supplementary Table S1). A 22.4 ms excitation with flyback gradients was applied (24), alternating between a 15-degree pulse at the pyruvate frequency and a 40-degree pulse at the lactate frequency, each followed by a single-shot spiral readout (40 × 40 points, 20 cm FOV, TR 2 seconds, time resolution 4 seconds).

In one patient (patient 1, Supplementary Table S1), images were acquired using a dynamic coronal-iterative decomposition with echo asymmetry and least-squares estimation (IDEAL) spiral chemical shift imaging sequence (25) and data were processed as described previously (11). Baseline data of this patient were included in a previous publication (11). In another patient (patient 3, Supplementary Table S1), only spectral data at a temporal resolution of 16 seconds were acquired at baseline due to a technical failure; the same scanning approach was repeated at follow up to allow direct comparison. Because of the low temporal resolution in this case, LAC/PYR was calculated on the basis of the summed spectra and this patient was excluded from any summed SNR analyses due to lack of imaging data and from apparent exchange rate constant for pyruvate–lactate exchange (*k*_PL_) analyses due to low temporal resolution. Although the ^13^C-MRI technique varied between patients, it was the same for the baseline and follow-up examination in each patient (no intra-patient variation) to compare measurements between time points for the purpose of response assessment.

Signal-to-noise ratios (SNR) for pyruvate and lactate summed over time and the summed lactate-to-pyruvate ratio (LAC/PYR) were calculated from the sum-of-squares (SOS) image reconstructions (summed SNR_PYR_, summed SNR_LAC_, LAC/PYR; Supplementary Table S2). *k*_PL_ was computed using singular value decomposition (SVD) image reconstructions (*k*_PL_; Supplementary Table S2; ref. 26). Further details regarding SOS and SVD reconstructions and analysis of ^13^C-images are provided in the Supplementary Materials.

### Statistical analysis

Statistical analyses were performed using R (version 3.6.3; R Foundation). Relationships between variables were assessed using Pearson's correlation, including the correlation coefficient *r*. Differences between measurements were compared using a two-sided Student *t* test (paired if measurements for the same patients at baseline were compared with the follow-up). *P* values below 0.05 were considered significant. No multiple testing correction was applied: Significant tests should be interpreted as exploratory rather than confirmatory.

### RNA sequencing

Biopsy samples were obtained within the Personalized Breast Cancer Program. For biopsy samples of patients included in this study, RNA was extracted from frozen tumor tissue sections obtained using the QIAGEN AllPrep DNA/RNA Mini Kit (catalog no. 80204; QIAGEN; Supplementary Materials). RNA quantification was performed using Qubit RNA BR (Invitrogen/Thermo Fisher Scientific catalog no: Q10211). Assessment of the RNA integrity number was performed using a TapeStation RNA ScreenTape (Agilent Technologies).

RNA sequencing libraries were constructed using the TruSeq Stranded Total RNA Gold library preparation kit (Illumina). The libraries were sequenced as paired-end reads (2 × 75 cycles) on a HiSeq2500 platform to give a mean coverage of 150×. Gene count data were postprocessed included normalization, scaling, and the correction of library preparation effects (Supplementary Materials). In brief, Salmon version 0.14.1 was used to estimate gene expression. The resulting estimated counts were corrected for library size and gene length. Potential library preparation bias was corrected for and further normalization was applied transforming the values to log_2_ counts for linear modeling. For correlations between RNA expression data and imaging data (LAC/PYR) in our cohort of patients with breast cancer, we only included patients with identical image acquisition (patients 1 and 3 were excluded, Supplementary Table S1).

### METABRIC data

The expression for relevant genes in the METABRIC cohort was normalized, as described previously (27). Log-intensities were standardized using z-scores. Follow-up data and relapse information from METABRIC were curated and processed as described recently (28). The smoothed scatterplots and Pearson correlation coefficients in Fig. 5 were computed between each pair of variables, and the cor.test function in base R was used to test the hypothesis of no correlation. Further details regarding survival analysis are provided in Supplementary Materials.

### Data and code availability

Transcriptomic data for those tumors included in our study imaged with hyperpolarized ^13^C-MRI are deposited at the European Genome-phenome Archive (https://ega-archive.org/datasets/EGAD00001008141). Imaging raw data and MATLAB scripts for data in this article can be obtained from radiology-13c-mri-breast@lists.cam.ac.uk.

## Results

Seven patients with a histopathological diagnosis of early-stage breast cancer were enrolled, that is, tumors confined to the breast, with or without locoregional lymph node involvement, but no distant metastatic disease. These included four patients with TNBC (ER and PR negativity defined as Allred score 0 to 3), three of which were invasive cancers of no specific type (IC NST) and one apocrine invasive cancer; and three HER2^+^ patients with breast cancer (two of which were ER^+^PR^+^, and one ER^−^PR^−^). All patients underwent standard-of-care NACT, patients with HER2^+^ breast cancer received dual anti-HER2 therapy in addition to chemotherapy, and two patients with TNBC received additional olaparib, a PARP1/2 inhibitor, as part of a clinical trial (PARTNER Trial, a randomized, phase II/III trial to evaluate the safety and efficacy of the addition of olaparib to platinum-based NACT in patients with breast cancer with TNBC and/or germline *BRCA1/2* mutations). After 5–7 cycles of neoadjuvant treatment, three patients demonstrated pCR and four patients non-pCR. Of the three patients with eventual pCR, there was one ER^−^PR^−^HER^+^ patient and two ER^−^PR^−^HER2^−^ patients (one with additional olaparib treatment and one without). Further details regarding patient and cancer characteristics and the prescribed treatments are provided in Supplementary Table S1.

**Figure 1.**
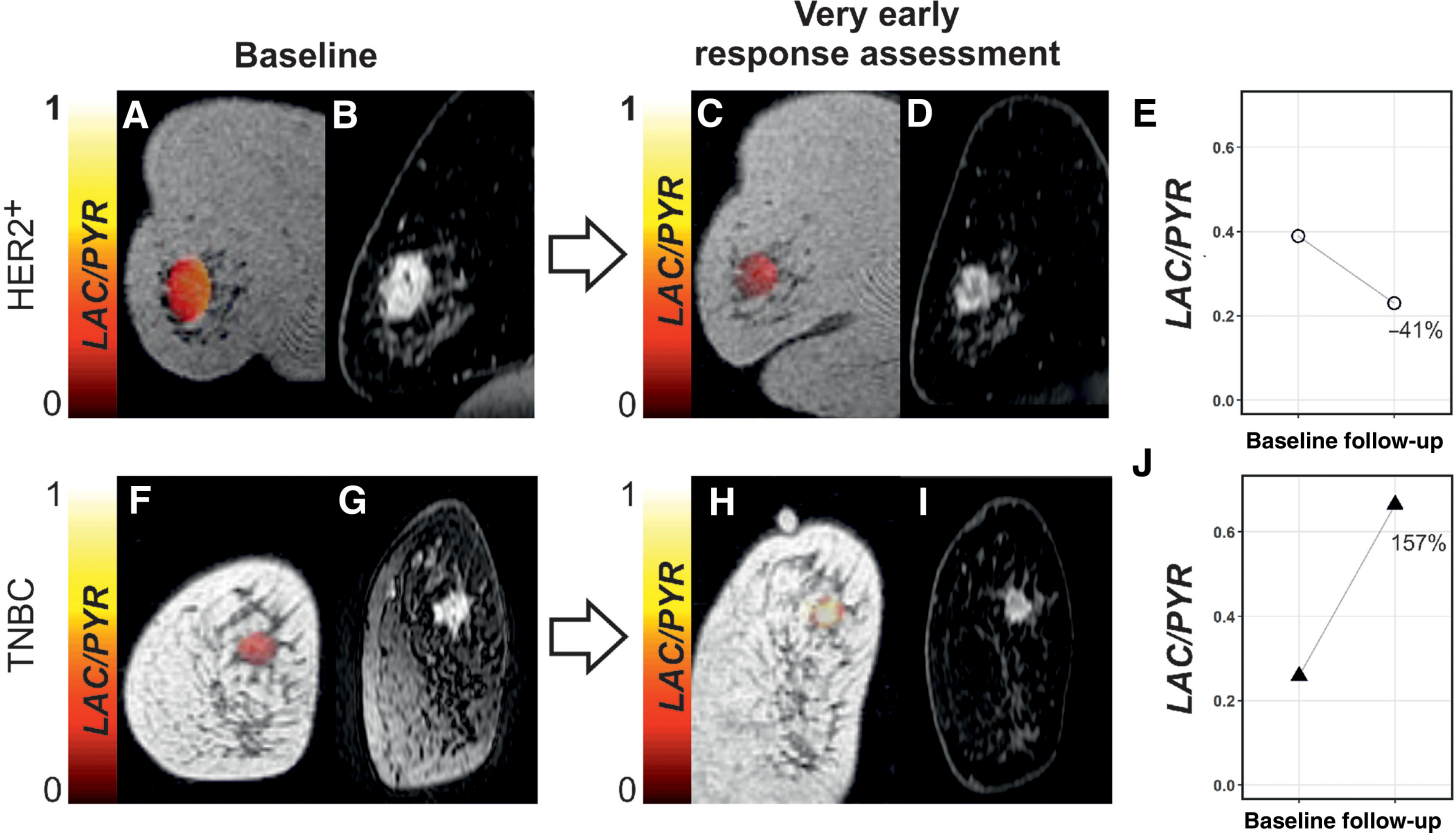
Changes in LAC/PYR between baseline and very early response assessment in a responder and nonresponder. **A**, **C**, **F**, and **H,** Coronal T_1_-weighted 3D spoiled gradient echo (SPGR) images with LAC/PYR map overlaid on the breast tumor. **B**, **D**, **G**, and **I,** Coronal reformatted DCE images obtained 150 seconds after intravenous injection of a gadolinium-based contrast agent. A patient with HER2^+^ breast cancer was imaged at baseline (**A** and **B**) and for ultra-early response assessment (**C** and **D**) following standard-of-care treatment and showed a decrease in LAC/PYR of 41% (**E**), indicating nonresponse. At surgery, non-pCR with residual invasive cancer was identified. Another patient with TNBC was imaged at baseline (**F** and **G**) and for ultra-early response assessment (**H** and **I**) following treatment with chemotherapy and a PARP inhibitor and showed an increase in LAC/PYR of 157% (**J**), indicating response. At surgery, pCR without residual invasive breast cancer was found. HER2^+^, HER2/neu positive.

Breast MRI was performed at baseline, before treatment initiation (median of 4 days between baseline ^13^C-MRI and treatment initiation), and as early follow-up for response assessment at 7–11 days after the first dose of NACT was administered (median = 7 days). Examples of one responder and one nonresponder imaged at these time points are shown in Fig. 1. Patient age at baseline was not significantly different between patients with eventual pCR and non-pCR (*P* > 0.05).

### ^13^C-MRI analysis

Mean values of ^13^C-MRI parameters computed for all voxels in each manually drawn region of interest (ROI) were analyzed. The number of voxels included in the ^13^C-MRI tumor ROIs ranged from 43 to 247 (mean = 102; median = 125). Results of the ^13^C-MRI and ^1^H-MRI data for all patients are shown in Supplementary Table S2.

**Figure 2.**
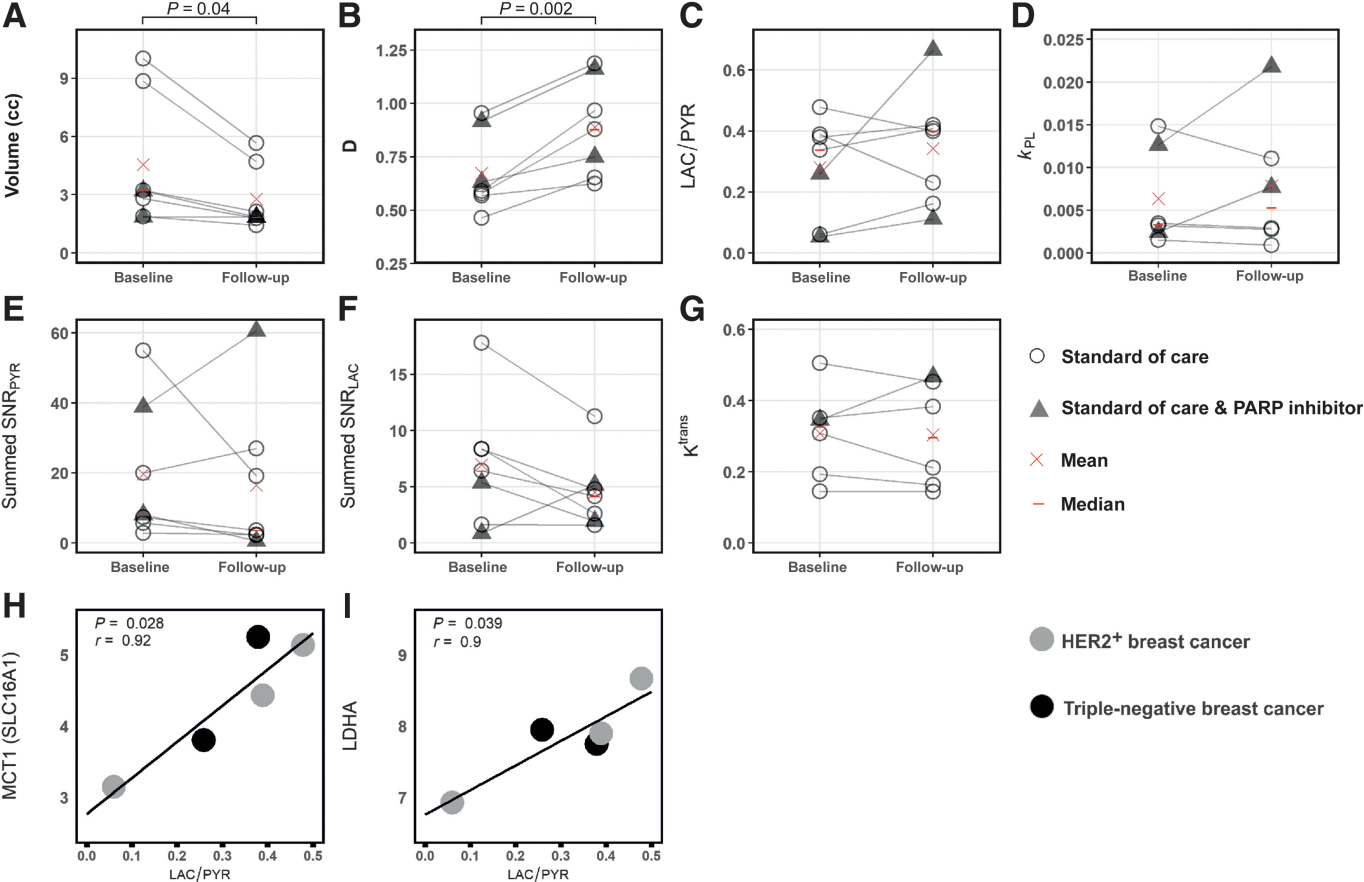
Parameters obtained from hyperpolarized ^13^C-MRI and ^1^H-MRI at baseline and in early follow-up scans. Differences between baseline and follow-up were significant for tumor volume (**A**) and diffusivity (**B**) but not for the other parameters (**C–G**); neither change in volume or diffusivity could distinguish pCR from non-pCR. Correlation of *SLC16A1* (MCT1) and *LDHA* mRNA expression with LAC/PYR was significant (**H** and **I**). Only images acquired with identical ^13^C-MRI acquisition parameters (spectral–spatial excitation) were included in these correlations.

**Figure 3.**
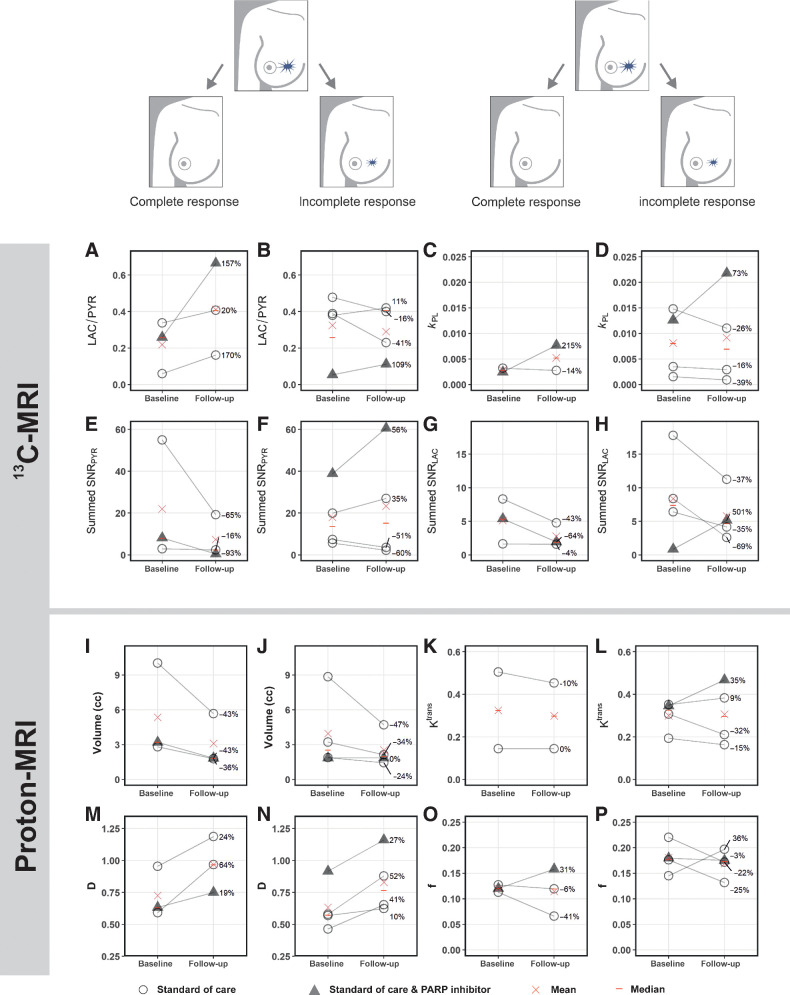
Changes in hyperpolarized ^13^C-, but not ^1^H-MRI–derived metrics, after approximately one week of treatment distinguish responders (pCR) from nonresponders (incomplete response; non-pCR). In the five patients undergoing standard-of-care neoadjuvant treatment, an increase of ≥20% in LAC/PYR was only observed in patients who responded (**A**), whereas a lower increase or even a decrease in LAC/PYR was observed in nonresponders (**B**).Both patients treated with a PARP inhibitor in addition showed an increase in LAC/PYR (**A** and **B**) and again the increase was highest in the responder (**A**). Although *k*_PL_ increased in all patients receiving a PARP inhibitor, but not in the other patients (**C** and **D**), neither *k*_PL_ nor any of the ^1^H-MRI–based metrics from dynamic contrast-enhanced (DCE) MRI (such as *K*^trans^) or from intravoxel incoherent motion (IVIM) as part of diffusion-weighted MRI (such as perfusion fraction *f* and tissue diffusivity *D*) could distinguish between responders and nonresponders (**I–P**). None of the parameters differed significantly between baseline and follow-up when evaluated for responders and nonresponders separately (*P* > 0.05). *k*_PL_ was not available in one patient due to technical failure (**C**). *K*^trans^ could not be assessed in one patient due to failed fat saturation (**K**).

**Figure 4.**
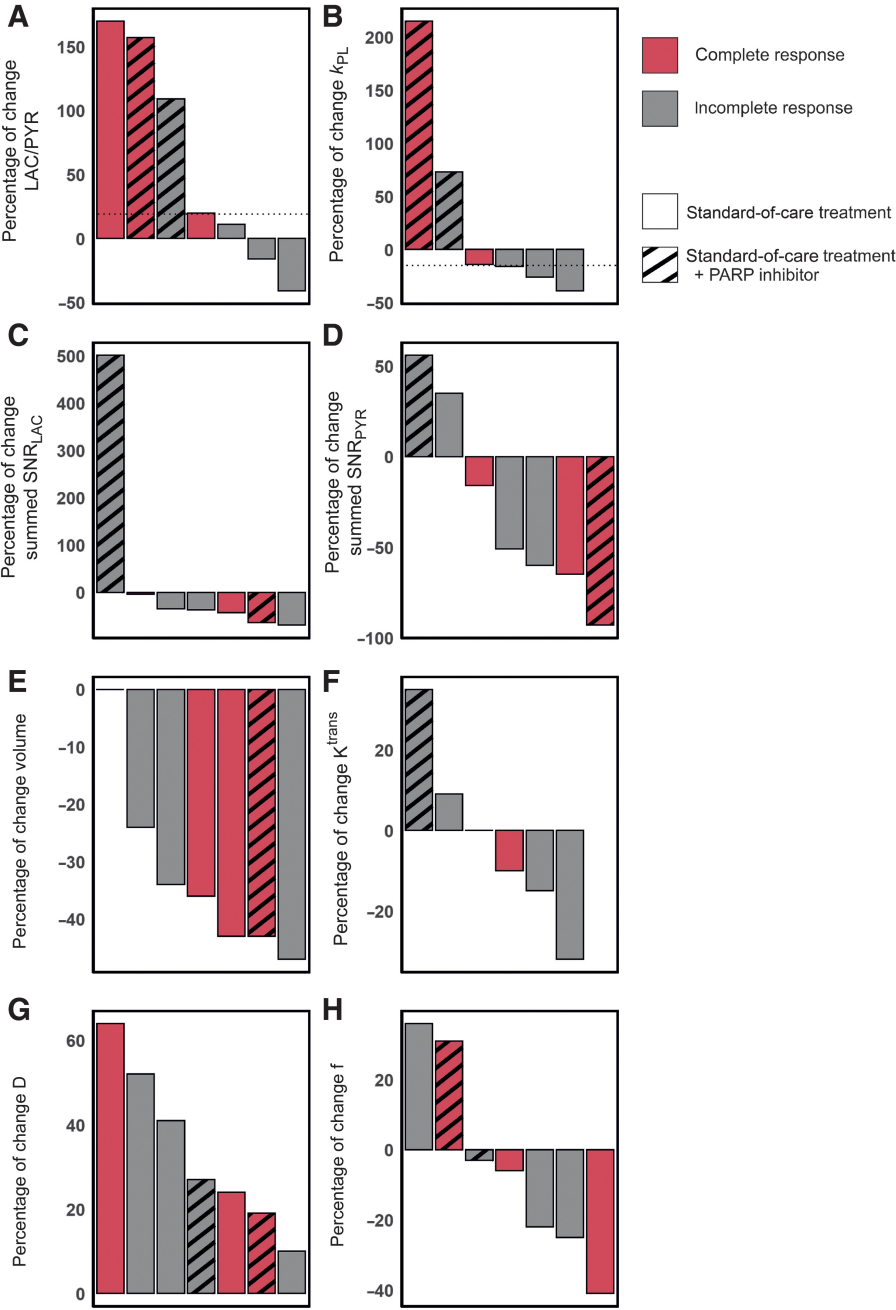
Changes in hyperpolarized ^13^C-MRI and ^1^H-MRI parameters in seven patients with complete and incomplete responses. Results are shown for standard-of-care treatment with and without PARP inhibitor treatment. **A,** A threshold of +20% change in LAC/PYR distinguished responders from nonresponders on standard-of-care therapy (shown with a dashed horizontal line). One nonresponder receiving PARP inhibitor treatment also showed an increase in LAC/PYR of ≥20%, which may be explained by NAD^+^ availability (see main text). **B,** A threshold set at a −15% change in *k*_PL_ (dashed horizontal line) distinguished responders from nonresponders on standard-of-care therapy. A patient receiving PARP inhibitor treatment in addition, but demonstrating pCR, also showed a change in *k*_PL_ above this threshold. *k*_PL_ was not available for one patient due to a technical failure. **C–H,** There were no thresholds that could be used to distinguish pCR from non-pCR for any of the remaining ^1^H-MRI or ^13^C-MRI parameters. Change in *K*^trans^ was not evaluable for one patient where fat saturation failed at baseline.

Mean summed SNR of pyruvate (summed SNR_PYR_) and lactate (summed SNR_LAC_) decreased between baseline and response assessment (summed SNR_PYR_ at baseline, mean ± SD = 19.7 ± 19.9; versus 16.5 ± 21.9 at response assessment; summed SNR_LAC_ at baseline 7.0 ± 5.6 versus 4.5 ± 3.3 at response assessment) whereas LAC/PYR and *k*_PL_ increased (LAC/PYR at baseline, mean ± SD = 0.28 ± 0.17 versus 0.34 ± 0.19 at response assessment; *k*_PL_ at baseline 0.0064 ± 0.0058 vs. 0.0079 ± 0.0078 at response assessment); however, these changes were not significant (*P* > 0.05). Results for these parameters and those derived from ^1^H-MRI are shown in Fig. 2 for the entire cohort, and for responders and nonresponders separately in Fig. 3. An increase of ≥20% in LAC/PYR measured using hyperpolarized ^13^C-MRI 7–11 days after commencing treatment, correctly predicted the three patients with eventual pCR at surgery. All patients with a change in LAC/PYR between baseline and early response assessment that was below a threshold of a 20% increase, demonstrated non-pCR at surgery (Fig. 4A). The only nonresponder with a change in LAC/PYR above this threshold received PARP inhibitor treatment in addition to standard therapy. For the two patients treated with a PARP inhibitor, the responder showed a higher increase in LAC/PYR than the nonresponder. A similar threshold of −15% for *k*_PL_ separated responders (above threshold) from nonresponders (below threshold), with the patient on PARP inhibition described above being the only nonresponder with an increase above this threshold (Fig. 4B). For the two patients receiving PARP inhibition, the increase in *k*_PL_ was again larger in the responder than the nonresponder. *k*_PL_ could not be calculated in one patient due to technical reasons. Changes in LAC/PYR and *k*_PL_ did not differ significantly between responders and nonresponders in this small cohort (*P* = 0.165 and *P* = 0.532, respectively). Neither changes in the summed SNR_LAC_ nor summed SNR_PYR_ could be used to distinguish between responders and nonresponders. Of note, changes in summed SNR_PYR_ may not only reflect biological changes such as perfusion and transport, but may also represent technical differences in polarization and coil sensitivity, and should therefore be interpreted with caution.

### ^1^H-MRI: volume, DCE, and diffusion-weighted imaging

HER2^+^ tumors were larger than TNBC at baseline (mean ± SD = 7.4 ± 3.6 mL and 2.4 ± 0.7 mL, respectively) and follow-up (4.2 ± 1.8 mL and 1.7 ± 0.2 mL, respectively), although this difference was not significant (*P* = 0.140 and *P* = 0.147, respectively). Tumor volume, as assessed on DCE-MRI, either decreased or remained stable in all tumors. Volume decreased significantly between baseline and response assessment (*P* = 0.04; Fig. 2A) in the whole cohort, but neither tumor volume at baseline (*P* > 0.05) nor volume changes could be used to distinguish responders and nonresponders (Fig. 4E).

No significant differences in the following DCE-MRI derived pharmacokinetic parameters were identified between tumors with pCR and non-pCR, or within either group between timepoints (*P* > 0.05): *K*^trans^ (the volume transfer constant as a measure of capillary permeability to gadolinium-based contrast agent that accumulates in the extravascular extracellular tumor compartment; Figs. 2G and 3K and L); *k*_ep_ (the rate constant reflecting transfer from the extravascular extracellular tumor compartment back into the plasma); *v*_e_ (volume of the extravascular extracellular space); and iAUC_90_ (integrated area under the enhancement-time curve 90 seconds after contrast injection). Change in *K*^trans^ was not evaluable for one patient where fat saturation failed at baseline.

The baseline perfusion fraction, *f*, measured using intravoxel incoherent motion (IVIM) was significantly lower in patients who reached pCR, compared with those with non-pCR (*P* = 0.026; Fig. 3O and P). The tissue diffusivity (*D*) increased significantly in all tumors between baseline and early response assessment (*P* = 0.002; Fig. 2B), whereas changes in *f* were variable. Importantly, changes in *f* and *D* were not significantly different between pCR and non-pCR tumors (*P* = 0.946 and *P* = 0.861, respectively) and could not be used to distinguish the two groups (Fig. 4G and H).

In the five patients with an identical ^13^C-MRI acquisition technique at both scanning timepoints, summed SNR_PYR_, summed SNR_LAC_, and LAC/PYR were significantly correlated with volume as assessed on DCE-MRI (*r* = 0.87, *P* = 0.001; *r* = 0.68, *P* = 0.030; and *r* = −0.63, *P* = 0.049, respectively; Supplementary Table S3; Supplementary Fig. S1). Summed SNR_PYR_ was more strongly correlated with volume than summed SNR_LAC_, resulting in a negative correlation of volume with LAC/PYR. *k*_PL_ was not significantly correlated with volume (*r* = 0.42, *P* = 0.231), but was positively correlated with the perfusion fraction *f* (*r* = 0.65, *P* = 0.044; Supplementary Fig. S1). Correlations between the hyperpolarized ^13^C-MRI parameters (summed SNR_PYR_, summed SNR_LAC_, LAC/PYR and *k*_PL_) and the DCE-MRI parameters (*K*^trans^, *k*_ep_, *v*_e_ and iAUC_90_) were low and non-significant (*P* > 0.05; Supplementary Table S3), indicating that they may be independent. The percentage changes in *k*_PL_ and LAC/PYR following treatment did not correlate with changes in volume or diffusion, showing that metabolic alterations were independent of size and cellularity. Percentage changes in *v*_e_ were significantly correlated with changes in LAC/PYR but not *k*_PL_ (*r* = 0.84, *P* = 0.035 and *r* = 0.14, *P* = 0.819, respectively). However, other DCE-MRI parameters such as *K*^trans^ and *k*_ep_ did not correlate with changes in metabolism. Importantly, changes in DCE-MRI, diffusion, and volume could not he used to correctly identify responders.

### RNA expression

The hyperpolarized ^13^C-MRI metrics LAC/PYR and *k*_PL_ were compared with RNA sequencing of biopsy samples at baseline, in those cases where ^13^C-MRI was acquired using an identical acquisition technique (*n* = 5). We have previously demonstrated that LAC/PYR correlates with the expression of the membrane transporter for pyruvate (monocarboxylic acid transporter 1, MCT1) in a cohort of treatment-naïve breast cancers (11). Here, we also observed a significant positive correlation between LAC/PYR at baseline and expression of the solute carrier 16A1 (*SLC16A1*), the gene encoding MCT1 (*r* = 0.92, *P* = 0.028; Fig. 2H), and in addition that LAC/PYR correlates with the expression of lactate dehydrogenase A (*LDHA*), the gene encoding a subunit of the enzyme LDH that catalyzes the exchange reaction between pyruvate and lactate (*r* = 0.90, *P* = 0.039; Fig. 2I). Correlations of *SLC16A1* (MCT1) and *LDHA* gene expression with *k*_PL_ were not significant (*P* = 0.984 and *P* = 0.924, respectively). LAC/PYR and *k*_PL_ were not significantly correlated with gene expression of carbonic anhydrase 9 (*CAIX*) and *HIF1A* (*P* > 0.05).

### Expression of *LDHA*, *SLC16A1* (MCT1), *HIF1A*, and *CAIX* and survival in different breast cancer subtypes

**Figure 5.**
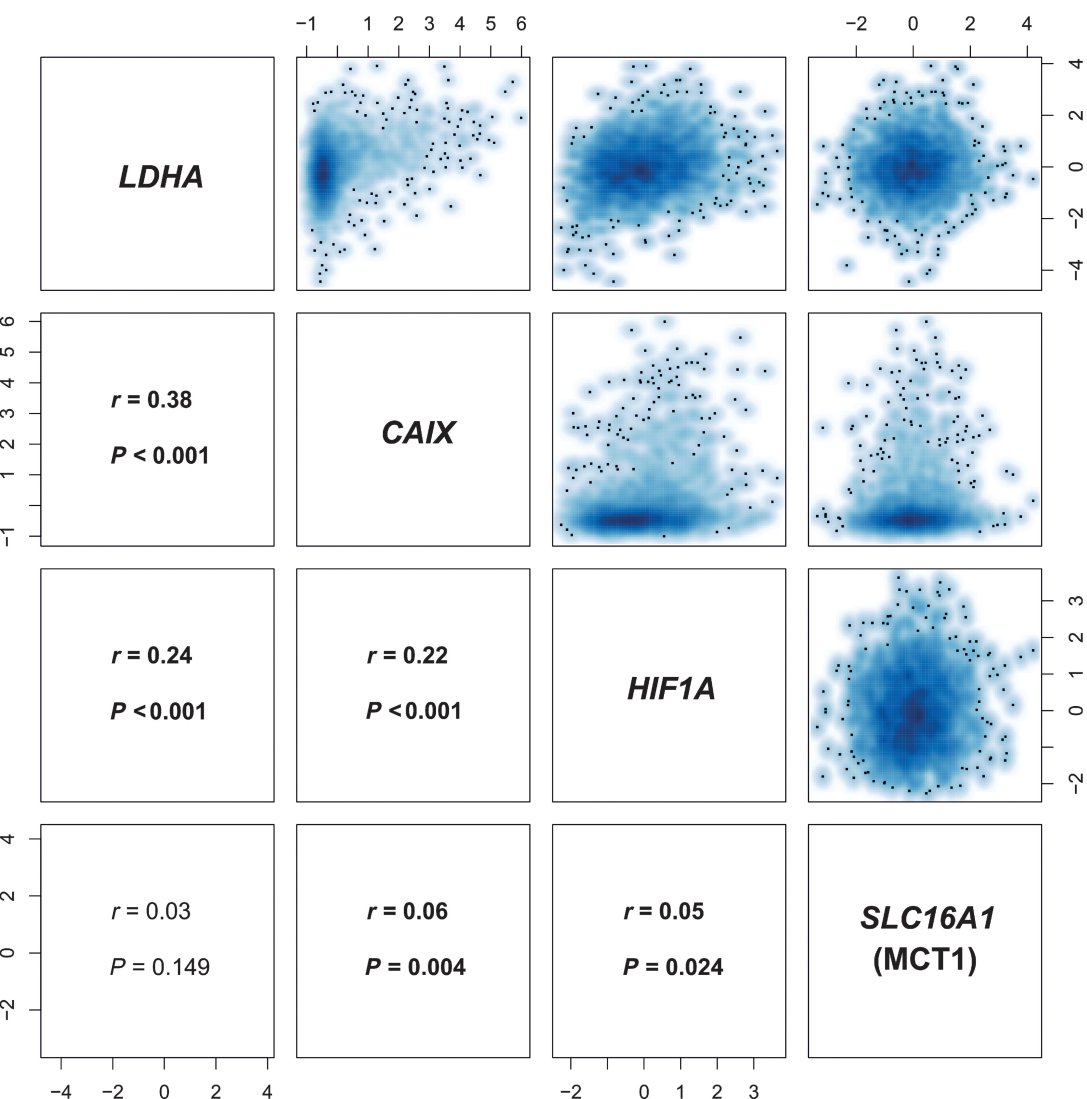
Correlation matrix of *LDHA*, *SLC16A1* (MCT1), *CAIX*, and *HIF1A* expression in METABRIC. There is a significant correlation between *LDHA* and *SLC16A1* (MCT1) expression (z-scores) with the hypoxia markers *CAIX* and *HIF1A*. *r*, Pearson correlation coefficient.

**Figure 6.**
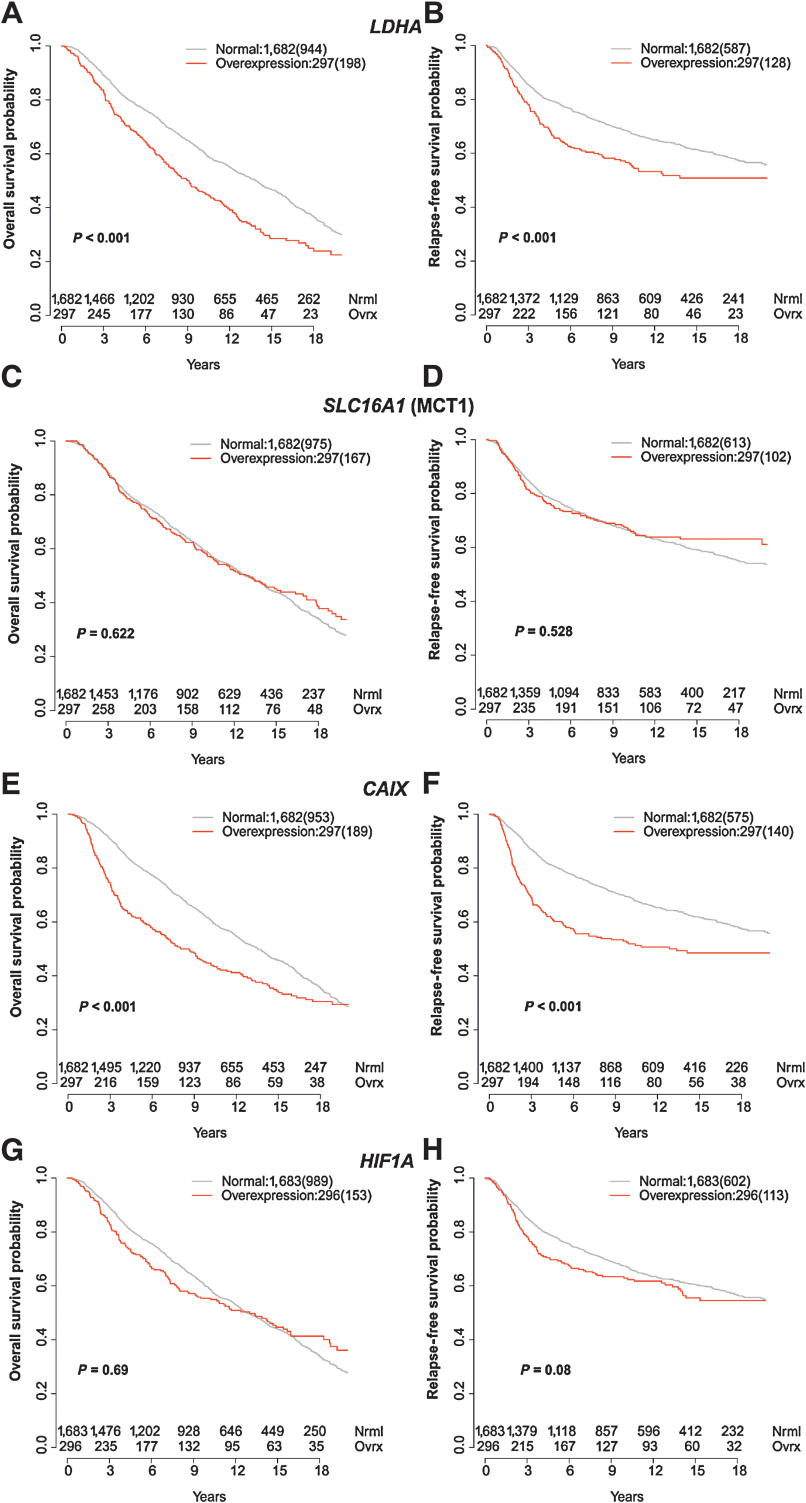
Correlation of *LDHA*, *SLC16A1* (MCT1), *CAIX*, and *HIF1A* expression with survival in METABRIC. Kaplan–Meier curves for normal expression and overexpression (85^th^ percentile) of *LDHA* (**A** and **B**), *SLC16A1* (MCT) (**C** and **D**), *HIF1A* (**E** and **F**), and *CAIX* (**G** and **H**). The left column shows overall survival and the right column relapse-free survival. Number of events are shown in brackets.

To further explore the significance of these findings, the intercorrelations of *LDHA*, *SLC16A1* (MCT1), *HIF1A*, and *CAIX* expressions were analyzed in a larger cohort, to evaluate their association with survival. Molecular Taxonomy of Breast Cancer International Consortium (METABRIC) is a large genomic and transcriptomic dataset acquired from approximately 2,000 breast cancer biopsy samples (27) with its outcome update published in 2019 (28). Although the expression of both, *LDHA* and *SLC16A1* (MCT1), was significantly correlated with expression of *HIF1A* and *CAIX*, the expression of *LDHA* was not significantly correlated with *SLC16A1* (MCT1) (Fig. 5). Overexpression of *LDHA* and the hypoxia marker *CAIX* [but not *SLC16A1* (MCT1) or *HIF1A*] was also associated with poorer overall survival (OS) and relapse-free survival (Fig. 6).

In addition, we also selected 501 patients from METABRIC whose breast cancer receptor status matched the patients in our imaged cohort (ER^−^PR^−^HER2^−^ = 320 patients; ER^−^PR^−^HER2^+^ = 134 patients; ER^+^PR^+^HER2^+^ = 47 patients). Although the expression of *LDHA* was significantly correlated with expression of *HIF1A* and *CAIX*, the expression of *SLC16A1* (MCT1) was not significantly correlated with either of the two in this subset of patients (Supplementary Fig. S2I). Overexpression of the hypoxia markers *CAIX* and *HIF1A* was associated with poorer OS and overexpression of CAIX was also associated with relapse-free survival (Supplementary Fig. S2A–S2H).

## Discussion

Most anticancer therapies induce metabolic alterations that precede the changes in tumor size that are measured routinely on standard-of-care imaging (29, 30). Therefore, noninvasive methods for imaging tumor metabolism offer the possibility for earlier detection of treatment response. Hyperpolarized ^13^C-MRI measurements using [1–^13^C]pyruvate as a metabolic probe have shown great promise for early therapy response monitoring in a large number of preclinical cancer models, including breast cancer. We and other groups have demonstrated how lactate labeling varies with tumor grade (11, 13). We have also shown that in breast cancer LAC/PYR correlates with both the expression of MCT1, which is responsible for the cellular uptake of pyruvate, and hypoxia as measured from HIF1α expression (11). To date, there have been two clinical case reports demonstrating a decrease in tumor lactate labeling following treatment in a TNBC and a high-grade prostate cancer after 3 weeks and 6 weeks of standard-of-care therapy, respectively (16, 31). The aim of this study was to evaluate the potential of hyperpolarized ^13^C-MRI to measure very early response to therapy in breast cancer, 7–11 days after initiation of treatment, and to compare it with ^1^H-MRI measures of vascular permeability using DCE-MRI and tissue cellularity using IVIM DWI.

The results show that an increase of ≥20% in LAC/PYR measured using hyperpolarized ^13^C-MRI 7–11 days after commencing standard-of-care treatment correctly predicted patients with eventual pCR at surgery. Changes in no other parameter, either using hyperpolarized ^13^C-MRI or advanced multiparametric ^1^H-MRI, could correctly identify patients with pCR. The threshold of 20% for determining response is in line with criteria such as RECIST or Positron Emission Tomography Response Criteria in Solid Tumors (PERCIST), where changes higher than 20% or 30% in size or metabolism, form the basis of determining tumor progression, response, or stable disease (30, 32). These results indicate that the technique holds promise for ultra-early response monitoring of NACT after 7–11 days of standard-of-care treatment, although this requires validation in larger cohorts.

The increase in LAC/PYR following treatment is interesting as the majority of preclinical hyperpolarized ^13^C-MRI studies have reported decreased lactate labeling following a positive response to therapy (16, 17, 20–22, 31, 33). However, a treatment-induced increase in lactate labeling has been shown in a smaller number of *in vitro* and *in vivo* models (18, 19, 34, 35). The accurate prediction of pCR with an increase in lactate labeling, rather than a decrease, raises important questions about the dynamic alterations in metabolism following treatment and the ideal timing for measuring a clinically meaningful metabolic response in future studies. Later timepoints may be dominated by opposing effects such as loss of cellularity, and this has important implications for the timing of treatment response monitoring, which are likely to be tumor- and treatment-specific.

There are a number of potential biological factors contributing to the changes in hyperpolarized ^13^C-label exchange between pyruvate and lactate following treatment (15). For example, the intracellular pyruvate concentration is determined by both tissue perfusion and membrane transport. Importantly, there were no significant differences in the DCE measures of vascularity (*K*^trans^, *v_e_*, or iAUC_90_) between baseline and the early treatment timepoint, suggesting that pyruvate delivery via the bloodstream alone cannot account for the changes in lactate labeling. Pyruvate transport is mediated by monocarboxylate transporters (MCT), of which, MCT1 and MCT4 are the most widely expressed in human tissue. MCT1 has a greater affinity for pyruvate and is the main transmembrane transporter for hyperpolarized [1–^13^C]pyruvate, which has been shown to be rate limiting for hyperpolarized [1–^13^C]lactate formation in some breast cancer cell lines and can account for treatment-induced changes (19, 36). We have previously demonstrated the importance of *SLC16A1* (MCT1) expression for determining lactate labeling in treatment-naïve breast tumors (11), which we have confirmed within this cohort. MCT1 has also been shown to have a role in lactate labeling in prostate cancer (13). Therefore, alterations in *SLC16A1* (MCT1) expression or its cellular localization could account for the changes in lactate labeling seen here following treatment. The absence of a significant change in vascular permeability or *K*^trans^ (Fig. 2G) following treatment suggests that vascular delivery of pyruvate is not responsible for the changes in LAC/PYR seen following treatment and further supports a potential role for MCT1 in these changes.

Intracellularly, the enzyme lactate dehydrogenase (LDH) catalyzes the conversion of pyruvate to lactate in the presence of the cofactor NADH. A reduction in LDH expression has been shown to mediate reduced lactate labeling in hyperpolarized ^13^C-MRI experiments (17, 22, 33), as has a decrease in NADH (20, 21, 37), and a decrease in the intracellular lactate pool (20, 34). Conversely, increasing lactate pool size and LDH expression have been shown to be associated with increasing lactate labeling after treatment (19). Here, we have shown that *LDHA* expression on an individual patient level, in addition to MCT1, correlates with the LAC/PYR ratio. Taken together with the previous data we have reported, these suggest that changes in *LDHA*, in addition to MCT1, may explain the after treatment changes we have shown here.

Hypoxia may be the driver for the relationship between LAC/PYR and both MCT1 and *LDHA*, as well as the changes in LAC/PYR seen after treatment. Tissue lactate is closely related to hypoxia and we have shown previously a correlation between *HIF1A* expression and LAC/PYR in a varied group of treatment-naïve breast cancers (11). Angiogenesis inhibitors have been shown to increase lactate labeling in a preclinical model of ovarian cancer assessed with hyperpolarized ^13^C-MRI (18). Antiangiogenesis is also a well-documented effect of taxanes (38) and all patients in our cohort received either weekly doses of paclitaxel or three-weekly docetaxel (Supplementary Table S1). In the absence of significant changes in the measured pharmacokinetic parameters (*K*^trans^, *v_e_*, or iAUC_90_) on DCE-MRI, any changes in hypoxia are likely to be driven by cellular changes in oxygen demand rather than by changes in the vasculature. Immune cell infiltration has been shown to contribute to the uptake of the radiolabelled glucose analog [^18^F]fluorodeoxyglucose (^18^F-FDG) on PET in patients with breast cancer following response to hormonal therapy (39, 40). An increase in tumor-infiltrating immune cells in responders could therefore also increase LAC/PYR more strongly as seen in our study. Future analysis of tumor tissue at this very early response assessment timepoint will further elucidate the mechanisms responsible for the increasing LAC/PYR in responding patients. Very early treatment-induced changes in cellular redox status could also contribute to these findings. For example, the early effects of treatment may generate a reducing environment with increasing NADH-favoring lactate formation, whereas a later oxidized state would increase the relative concentration of NAD^+^ favoring conversion from lactate-to-pyruvate at later stages of treatment (41). However, tissue redox status is difficult to assess in practice, particularly at multiple timepoints during treatment but future validation of our results with assays quantifying NAD^+^ (and protein expression of MCT1, LDHA, CAIX, and HIF1A) will be important to fully understand the molecular changes underlying our imaging results.

To validate some of our findings in an external cohort, and to understand the clinical significance of the results, we subsequently analyzed the expression data from almost 2,000 patients in the METABRIC dataset (27, 28). In addition to *SLC16A1* (MCT1) and *LDHA*, *CAIX* was assessed as a stable membrane-bound isoform of the enzyme carbonic anhydrase that is upregulated in hypoxic conditions and has been shown to be important for pH regulation of tumors (42). *LDHA* and *SLC16A1* (MCT1) expressions were significantly correlated with expression of both hypoxia markers, *CAIX* and *HIF1A*, showing the important interrelationship between pyruvate metabolism, lactate formation, and hypoxia. There was a strong correlation between overexpression of both *LDHA* and *CAIX* and a statistically significant reduction in relapse-free survival and OS. Therefore, LAC/PYR may provide important prognostic information in addition to detection of early treatment response. It also suggests the importance of combining imaging measures of hypoxia and metabolism with histopathological metrics to more fully phenotype tumors, as well as for prognosis.

Olaparib inhibits PARP1 and 2, two enzymes functioning as DNA damage sensors and facilitators of DNA damage repair mechanisms by using NAD^+^ as a substrate to PARylate themselves and other target proteins (43, 44). Although up to 90% of cellular NAD^+^ can be used by PARPs in response to cellular DNA damage, PARP inhibitors compete with NAD^+^ for the catalytic cages of PARPs and trap PARPs at DNA double-strand breaks to impair PARylation and the DNA damage repair it mediates (44–46). Both patients receiving a PARP inhibitor in addition to standard-of-care treatment (one with eventual pCR and another one with non-pCR) also demonstrated an increase in LAC/PYR greater than 20%, and the increase in LAC/PYR was much higher in the patient with pCR than non-pCR, again pointing toward a greater increase in responders. PARP inhibitors have been shown to increase NAD^+^ levels in both murine cells and liver tissue (47), and decreasing NAD^+^ levels have been correlated with decreasing lactate labeling in hyperpolarized ^13^C-MRI experiments previously (20,21,37). These results suggest that increasing availability of intracellular NAD(H) due to PARP inhibitor treatment may elevate LDH-mediated lactate labeling. PARP inhibitors might, therefore, cause a pharmacodynamic increase in LAC/PYR and *k*_PL_ regardless of response, and the larger increase in the patient who responded may be accounted for by additional changes in MCT1 and/or LDHA, or an increase in immune cell infiltration. A previous study in the MCF-7 breast cancer cell line has shown that lactate increases with an MEK inhibitor, which was explained by activation of the PI3K and/or AMPK pathways (34). These results suggest that caution is warranted in interpreting results in patients undergoing experimental targeted treatment where the mode of action could influence lactate production.

Pharmacokinetic parameters from DCE-MRI and diffusivity from DWI (including IVIM) have also been explored for early response prediction in breast cancer, with *K*^trans^ typically decreasing and diffusivity increasing in responders compared with nonresponders (48–50). However, in most studies these assessments are made later during NACT than in this study. Our data showed that the perfusion fraction, *f*, determined using IVIM differs between pCR and non-pCR even at baseline in this cohort. A previous study found significant differences in baseline measurements of *f* in patients with eventual pCR when compared with those with non-pCR, although *f* was higher in patients with pCR in their cohort, which contrasts with our results (48). The different composition of the cohorts may account for this difference given the inclusion of mainly ER^+^ breast cancers previously. As with the findings from hyperpolarized ^13^C-MRI, these IVIM-derived metrics need validation in larger cohorts. Also, diffusivity *D* was not found to predict pathological response in our cohort, indicating that there was no significant change in cellularity that could account for the alterations in lactate labeling at this early treatment response timepoint.

We also showed that LAC/PYR is the most reliable metric to distinguish between responders and nonresponders undergoing standard-of-care treatment in our study. Results for *k*_PL_ were similar, although there was a smaller difference between responders and nonresponders and fewer available data. As the estimation of *k*_PL_ depends on the SNR in images acquired at single time points, it is less accurate at lower SNR than LAC/PYR, which is calculated from the time-summed images. Our results suggest that LAC/PYR may be the most precise metric for use in the clinical setting to assess a 20% change following treatment.

The limitations of our study include the small size of the dataset. The preliminary findings arising from this work will need to be confirmed and validated in larger cohorts, ideally as part of large multisite studies. However, this was a prospective study and we included patients with the two most common breast cancer subtypes who frequently undergo NACT: TNBC and HER2^+^. Despite the small group size, we show in both groups undergoing standard-of-care treatment that an increase of LAC/PYR of at least 20% indicates pCR and that in both the pCR and non-pCR groups there was one patient treated with a PARP inhibitor in addition. We have explored the significance of this small dataset by analyzing the very large expression dataset within METABRIC that has shown the potential importance of the technique in demonstrating prognosis.

In conclusion, an increase in the LAC/PYR ratio of ≥20%, measured using hyperpolarized ^13^C-MRI, has the potential to distinguish between responding and nonresponding patients with breast cancer undergoing standard-of-care neoadjuvant treatment as early as 7–11 days after the start of treatment. LAC/PYR is significantly correlated with both *LDHA* and *SLC16A1* (MCT1) expressions in this cohort. Comparison with a large breast cancer gene expression dataset (METABRIC) showed that *LDHA* and *SLC16A1* (MCT1) expressions are significantly correlated with expression of the hypoxia markers *CAIX* and *HIF1A* and that overexpression of the hypoxia markers *CAIX* and *LDHA* is significantly associated with shorter OS and relapse-free survival in breast cancer.

## Authors' Disclosures

R. Woitek reports grants from AstraZeneca and Cancer Research UK (CRUK), Illumina and CRUK, Austrian Science Fund, and non-financial support from GE Healthcare, and grants from Mark Foundation for Cancer Research, Cancer Research UK Cambridge Center (grant C9685/A25177), Cancer Research UK (CRUK; grants C8742/A18097, C19212/A16628, C19212/A911376, and C197/A16465), CRUK Cambridge Center, the CRUK and Engineering and Physical Sciences Research Council Cancer Imaging Center in Cambridge and Manchester, Addenbrooke's Charitable Trust, and the NIHR Cambridge Biomedical Research Center, and non-financial support from Cambridge Breast Cancer Research Unit Laboratory during the conduct of the study. L. Beer reports grants from AstraZeneca and Cancer Research UK (CRUK), Illumina and CRUK, The Mark Foundation for Cancer Research, Cancer Research UK Cambridge Center, Cancer Research UK, and CRUK Cambridge Center, the CRUK and Engineering and Physical Sciences Research Council Cancer Imaging Center in Cambridge and Manchester, Addenbrooke's Charitable Trust, and the NIHR Cambridge Biomedical Research Center, and non-financial support from Cambridge Breast Cancer Research Unit Laboratory and GE Healthcare during the conduct of the study. G. Baxter reports grants from AstraZeneca and Cancer Research UK (CRUK), Illumina and CRUK, The Mark Foundation for Cancer Research, Cancer Research UK Cambridge Center, Cancer Research UK, and CRUK Cambridge Center, the CRUK and Engineering and Physical Sciences Research Council Cancer Imaging Center in Cambridge and Manchester, Addenbrooke's Charitable Trust, and the NIHR Cambridge Biomedical Research Center, and non-financial support from Cambridge Breast Cancer Research Unit Laboratory and GE Healthcare during the conduct of the study. L. Rundo reports personal fees from Lucida Medical Ltd. outside the submitted work. E. Provenzano reports grants from AstraZeneca and Cancer Research UK (CRUK), Illumina and CRUK, The Mark Foundation for Cancer Research, Cancer Research UK Cambridge Center, CRUK Cambridge Center, the CRUK and Engineering and Physical Sciences Research Council Cancer Imaging Center in Cambridge and Manchester, Addenbrooke's Charitable Trust, and the NIHR Cambridge Biomedical Research Center, non-financial support from Cambridge Breast Cancer Research Unit Laboratory, and non-financial support from GE Healthcare, and personal fees from NIHR Cambridge Biomedical Research Center during the conduct of the study; personal fees and non-financial support from Roche Pharmaceuticals outside the submitted work. J.D. Kaggie reports grants from National Institute of Health Research (NIHR) Cambridge during the conduct of the study; as well as grants from GlaxoSmithKline and grants from EU Horizon 2020 outside the submitted work. A. Frary reports grants from AstraZeneca and Cancer Research UK (CRUK), Illumina and CRUK, The Mark Foundation for Cancer Research, Cancer Research UK Cambridge Center, Cancer Research UK, CRUK Cambridge Center, the CRUK and Engineering and Physical Sciences Research Council Cancer Imaging Center in Cambridge and Manchester, Addenbrooke's Charitable Trust, and the NIHR Cambridge Biomedical Research Center, and non-financial support from Cambridge Breast Cancer Research Unit Laboratory and GE Healthcare during the conduct of the study. J. Kane reports grants from AstraZeneca and Cancer Research UK (CRUK), Illumina and CRUK, The Mark Foundation for Cancer Research, Cancer Research UK Cambridge Center, Cancer Research UK, CRUK Cambridge Center, the CRUK and Engineering and Physical Sciences Research Council Cancer Imaging Center in Cambridge and Manchester, Addenbrooke's Charitable Trust, and the NIHR Cambridge Biomedical Research Center, non-financial support from Cambridge Breast Cancer Research Unit Laboratory and GE Healthcare during the conduct of the study. A.J.V. Benjamin reports grants from AstraZeneca and Cancer Research UK (CRUK), Illumina and CRUK, The Mark Foundation for Cancer Research, Cancer Research UK Cambridge Center, Cancer Research UK, CRUK Cambridge Center, the CRUK and Engineering and Physical Sciences Research Council Cancer Imaging Center in Cambridge and Manchester, Addenbrooke's Charitable Trust, and the NIHR Cambridge Biomedical Research Center, and non-financial support from Cambridge Breast Cancer Research Unit Laboratory and GE Healthcare during the conduct of the study; as well as grants from National Cancer Imaging Translational Accelerator outside the submitted work. A.N. Priest reports personal fees from GE Healthcare outside the submitted work. B. White reports grants from AstraZeneca and Cancer Research UK (CRUK), Illumina and CRUK, The Mark Foundation for Cancer Research, Cancer Research UK Cambridge Center, Cancer Research UK, CRUK Cambridge Center, the CRUK and Engineering and Physical Sciences Research Council Cancer Imaging Center in Cambridge and Manchester, Addenbrooke's Charitable Trust, and the NIHR Cambridge Biomedical Research Center, and non-financial support from Cambridge Breast Cancer Research Unit Laboratory and GE Healthcare during the conduct of the study. B. Carmo reports grants from AstraZeneca and Cancer Research UK (CRUK), Illumina and CRUK, The Mark Foundation for Cancer Research, Cancer Research UK Cambridge Center, Cancer Research UK, CRUK Cambridge Center, the CRUK and Engineering and Physical Sciences Research Council Cancer Imaging Center in Cambridge and Manchester, Addenbrooke's Charitable Trust, and the NIHR Cambridge Biomedical Research Center, as well as grants and non-financial support from Cambridge Breast Cancer Research Unit Laboratory, and non-financial support from GE Healthcare during the conduct of the study. R. Slough reports grants from AstraZeneca and Cancer Research UK (CRUK), Illumina and CRUK, The Mark Foundation for Cancer Research, Cancer Research UK Cambridge Center, Cancer Research UK, CRUK Cambridge Center, the CRUK and Engineering and Physical Sciences Research Council Cancer Imaging Center in Cambridge and Manchester, Addenbrooke's Charitable Trust, and the NIHR Cambridge Biomedical Research Center, and non-financial support from Cambridge Breast Cancer Research Unit Laboratory and GE Healthcare during the conduct of the study. R.F. Schulte reports other support from GE Healthcare during the conduct of the study; and other support from GE Healthcare outside the submitted work. S. Chin reports grants from AstraZeneca and Cancer Research UK (CRUK), Illumina and CRUK, The Mark Foundation for Cancer Research, Cancer Research UK Cambridge Center, Cancer Research UK, CRUK Cambridge Center, the CRUK and Engineering and Physical Sciences Research Council Cancer Imaging Center in Cambridge and Manchester, Addenbrooke's Charitable Trust, and the NIHR Cambridge Biomedical Research Center, and non-financial support from Cambridge Breast Cancer Research Unit Laboratory and GE Healthcare during the conduct of the study. M. Graves reports grants from AstraZeneca and Cancer Research UK (CRUK), Illumina and CRUK, The Mark Foundation for Cancer Research, Cancer Research UK Cambridge Center, Cancer Research UK, CRUK Cambridge Center, the CRUK and Engineering and Physical Sciences Research Council Cancer Imaging Center in Cambridge and Manchester, Addenbrooke's Charitable Trust, and the NIHR Cambridge Biomedical Research Center, non-financial support from Cambridge Breast Cancer Research Unit Laboratory, GE Healthcare, and Rapid Biomedical during the conduct of the study; and personal fees from GE Healthcare and Bayer outside the submitted work. F.J. Gilbert reports grants from AstraZeneca and Cancer Research UK (CRUK), Illumina and CRUK, The Mark Foundation for Cancer Research, Cancer Research UK Cambridge Center, Cancer Research UK, NIHR Cambridge Biomedical Research Center, and non-financial support from GE Healthcare during the conduct of the study; and President European Society of Breast Imaging. J. Abraham reports grants from AstraZeneca and other support from Illumina during the conduct of the study; grants from AstraZeneca and other support from Illumina outside the submitted work. C. Caldas reports grants from AstraZeneca, Cancer Research UK, and Illumina during the conduct of the study; grants from AstraZeneca and Cancer Research UK outside the submitted work; and research grants administered by the University of Cambridge: AstraZeneca, Roche, Servier, Genentech, Varsity Therapeutics; and is a member of external science panel of AstraZeneca iMED and Illumina SAB. K.M. Brindle reports other support from GE Healthcare during the conduct of the study; as well as has a patent for WO2008020764 A1 issued. E. Sala reports grants from AstraZeneca and Cancer Research UK (CRUK), Illumina and CRUK, The Mark Foundation for Cancer Research, Cancer Research UK Cambridge Center, Cancer Research UK, CRUK Cambridge Center, the CRUK and Engineering and Physical Sciences Research Council Cancer Imaging Center in Cambridge and Manchester, Addenbrooke's Charitable Trust, and the NIHR Cambridge Biomedical Research Center, grants and non-financial support from Cambridge Breast Cancer Research Unit Laboratory, and GE Healthcare during the conduct of the study; personal fees from GlaxoSmithKline and other support from Lucida Medical outside the submitted work. F.A. Gallagher reports grants from AstraZeneca/CRUK, Illumina/CRUK, CRUK Cambridge Center, CRUK, NIHR Cambridge Biomedical Research Center, and non-financial support from Cambridge Breast Cancer Research Unit Laboratory, and GE Healthcare during the conduct of the study; as well as grants from GlaxoSmithKline, non-financial support from GE Healthcare, and other support from AstraZeneca outside the submitted work. No disclosures were reported by the other authors.

## Supplementary Material

Supplementary Materials Supplementary Materials (clean)
