# Supplementary material for: Hyperpolarized Carbon-13 MRI for Early Response Assessment of Neoadjuvant Chemotherapy in Breast Cancer Patients
Source: Cancer Res. 2021 Oct 8;81(23):6004–17. doi: 10.1158/0008-5472.CAN-21-1499 (PMC7612070; doi:10.1158/0008-5472.CAN-21-1499)
Supplement: Supplementary Materials — (clean) [file can-21-1499_supplementary_materials_suppsm1.pdf]

Supplementary Materials for: *Hyperpolarized carbon-13 MRI for early response assessment of neoadjuvant chemotherapy in breast cancer patients*

Supplementary Methods

^{1}H-MRI

Sequence specifications for T1-weighted axial and coronal 3D fast spoiled gradient echo images used to plan the ^{13}C-MRI:

FOV = 35 cm; matrix = 256 × 256 pixels; TE = 2.1 ms; TR = 5.292 ms; flip angle = 10°; slice thickness = 2.8 mm.

Dynamic contrast-enhanced (DCE) MRI was acquired using a 3D fast spoiled gradient echo sequence with *k*-space data sharing (volume image breast assessment–time-resolved imaging of contrast kinetics [VIBRANT-TRICKS]). Images were acquired with a repetition time (TR) of 7.1 ms, echo time (TE) of 3.8 ms, an in-plane voxel size of 0.68 × 0.68 mm^2, a slice thickness of 1.4 mm, a field-of-view of 350 mm, a matrix of 512 × 512 pixels, a flip angle of 12°, and fat suppression using spectral-spatial water excitation. Forty-eight VIBRANT-TRICKS volumes were acquired over 8 minutes, with a temporal resolution of 9.4 s. Contrast agent injection was started between the acquisition of volumes blocks two and three. Gadobutrol (Gadovist; Bayer Healthcare, Berlin, Germany) was injected at 0.1 mmol per kg of body weight and a flow rate of 3.0 mL/s followed by a 25 mL saline flush. Postprocessing of DCE-MRI is described in Supplementary Materials.

Postprocessing of DCE-MRI data: $T1_0$ maps were used to convert from signal intensity to gadolinium concentration. $T1_0$ mapping was based on a 3D variable flip angle (VFA) gradient echo method using FAs of 2°, 3°, 5°, 10°, and 15° used with an axial three-dimensional fast spoiled gradient-echo sequence (field of view, FOV = 350 mm; matrix = 256 × 256 pixels; interpolated slice thickness = 1.4 mm; in-plane voxel size = 0.6 × 0.6 mm²; TR = 5.3 ms; TE = 2.1 ms) (1). $T1_0$ maps were calculated using MIStar (Apollo Medical Imaging, Melbourne, Australia). RF transmit uniformity ($B1^+$) maps were obtained using a multi-slice 2D Bloch-Siegert–based gradient-echo sequence (2) with the images postprocessed using in-house code (MATLAB, The MathWorks, Inc., Natick, MA, USA); FOV = 350 mm; matrix = 128 × 128; slice thickness = 7 mm; in-plane voxel size = 2.7 × 2.7 mm²; TR = 29 ms; TE = 13.5 ms; FA = 20°. Spatial registration of $B1^+$ corrected $T1_0$ maps and DCE was adjusted manually where necessary.

For volumetry and the extraction of pharmacokinetic parameters from the DCE-MRI, tumor volumes of interest were drawn manually on the DCE-MRI data (images acquired around 160 s after contrast injection) by an attending radiologist (R.W.) with 10 years of radiologic experience who specialized in breast imaging (3D Slicer; https://www.slicer.org;(3)). These volumes of interest were used to extract the following pharmacokinetic parameters: transfer constant (K^{trans}), washout parameter (k_{ep}), extravascular extracellular volume (v_e), and area under the contrast concentration versus time curve 90 seconds after contrast material injection ($iAUC_{90}$). Pharmacokinetic modelling of the DCE-MRI data was undertaken using the software MIStar (Apollo Medical Imaging Technology, Melbourne, Australia), the Tofts model and a population based arterial input function (4,5). Mean

values of the pharmacokinetic parameters were calculated for all volumes of interest and only voxels with a high enough goodness of fit ($r^2 \geq 0.75$) were included in the analyses. In patient 6, failed fat saturation during the DCE-MRI did not allow reliable pharmacokinetic modelling.

IVIM was performed utilizing a single-shot spin-echo echo planar imaging sequence at eight b values (0, 30, 60, 90, 120, 300, 600 and 900 s/mm^2) as described previously (6). IVIM data were analysed using in-house software developed in MATLAB (The Mathworks, Natick, MA). Parametric maps of IVIM parameters (D, f and D^*) were generated using a non-linear least square fitting on a voxel-wise basis using all b values. Breast tumors were outlined for this analysis on the highest b value images using OsiriX (V8.5.2 Pixmeo SARL; OsiriX) and parameters were expressed as means over the volumes measured.

^{13}C-MRI

Pharmacy kits and samples were prepared, hyperpolarized, dissolved and assessed for pharmaceutical quality and suitability for injection as described previously (7,8). In brief, pharmacy kits were prepared containing 1.47 g of [1-^{13}C]-pyruvic acid and 15 mmol/L of an electron paramagnetic agent, then hyperpolarized, and rapidly dissolved using 38 mL of superheated sterile water. Suitability for injection was confirmed after filtration of the electron paramagnetic agent to less than or equal to 3 µM and buffered to adjust pH to between 6.0 to 8.2. Hyperpolarized pyruvate solution (0.4 mL/kg at a concentration of approximately 250 mmol/L) was injected at a rate of 5 mL/sec followed by a 25 mL saline flush. For ^{13}C MRI, a dedicated eight-channel ^{13}C breast coil (RAPID Biomedical, Rimpar, Germany) was used with a phantom containing a ^{13}C-labeled 8 mol/L urea sample (Sigma-Aldrich, St Louis, MO, USA) positioned adjacent to the tumor-containing breast.

We have found that sum-of-squares (SOS) image reconstructions are better suited for summed metabolite SNR and lactate-to-pyruvate ratio (SNR_{PYR}, SNR_{LAC}, LAC/PYR) while singular value decomposition (SVD) reconstructions are best for single time point SNR and k_{PL} estimation ($SNR_{PYR\ SVD}$, $SNR_{LAC\ SVD}$, LAC/PYR_{SVD}, k_{PL}). Therefore, our analyses are focusing on SOS and SVD for the indicated purposes. The SOS reconstruction weighted the contribution from each individual coil by its estimated peak signal of lactate divided by the square of the noise, where noise was taken to be the standard deviation of lactate signal in the image at the final timepoint, when the signal had decayed.

Elliptical ROIs for quantitative analysis of ^{13}C-images were drawn manually in ImageJ (9) taking into account the maximum transverse and cranio-caudal diameter of the main mass of enhancing tumor, but leaving out very fine spiculations. Coronal ^{13}C images, as well as coronal T1 3D SPGR images, acquired using the inbuilt body coil when the patient was positioned in the ^{13}C-tuned coil and coronally reformatted VIBRANT-TRICKS images acquired approximately 60 s after contrast administration were taken into account and displayed side by side to establish those ROIs to be used on the ^{13}C maps showing the largest tumor diameter for quantitative analysis. Mean values of the ^{13}C-metrics were calculated for all voxels included in an ROI.

RNA extraction

RNA from frozen tumor tissue sections was extracted using the QIAGEN AllPrep DNA/RNA Mini Kit cat no: 80204). Briefly, tissue sections were resuspended in 600ul of RLT buffer plus and homogenised using TissueLyser (Qiagen) for 2 min at 25Hz. (QIAGEN MaXtract, Venlo, Netherlands; Cat no: 129056). The samples were centrifuged at 12,000 g for 3 min at 4°C. The supernatant is transferred to a DNeasy column and centriuged at 8000 x g for 30s. DNA is extracted from the

DNeasy column. The eluate collected in the collection tube is mixed well with 600µl of 70% ethanol and transferred to a RNeasy column (several times if necessary) followed by centrifugation at 8000 x g for 30s. Wash column with 350µl of buffer RW1 and centrifuge for 15s at 8000 x g to wash the membrane. Add 80 µl DNase I incubation mix (10µl DNAse I and 70 µl RDD buffer) directly to the RNeasy spin column membrane and incubate at room temperature (20-30°C) for 15 minutes. After the incubation, add 350µl buffer RW1 to the RNeasy spin column, and centrifuge for 15s at 8000 x g. Discard the eluate and add 500µl Buffer RPE to the RNeasy spin column, and centrifuge for 15s at 8000 x g. Repeat the RPE buffer wash step and centrifuge for 2 min at 8000 x g. Discard the eluate and centrifuge the RNeasy spin column at full speed for 1 min to dry the membrane. Elute the RNA with 55µl of RNase free water and centrifuge for 1 min at 8000 x g. RNA is quantified using Qubit RNA Broad Range (Invitrogen/ThermoFisher Scientific catalog no: Q10211). Assessment of the RNA quality was performed using a TapeStation RNA ScreenTape (Agilent Technologies).

Postprocessing of RNA sequencing data.

To estimate gene counts we employed Salmon version 0.14.1 on read-based mode (10). The final estimated number of reads mapping to each gene was then normalized in three post-processing steps. First, we applied the *edgeR* package to estimate effective library sizes that account for sequencing depth and RNA composition (11). Second, to scale the normalized count data for linear modelling, the counts were transformed into log2 counts per million and assigned proper weights using the voom method in the *limma* package (12). Third, technical effects related to differences in library preparation were removed using ComBat from the *sva* package (13). Final values were also corrected for effective gene length.

METABRIC data

Figure 5, panels A-H were obtained using Kaplan-Meier estimates for overall survival and relapse-free survival according to the expression of each gene split into two groups defined by the 85th percentile. Differences in the survival curves were tested with the log-rank test. These computations were obtained using the survival R package (14) and the plots were produced with the 'rms' R package (15).

Breast density assessment

Breast parenchymal density was assessed according to the ACR BI-RADS Atlas on mammograms acquired at the time of assessment (16).

Supplementary Table 1. Patient details. All cancers were symptomatic and the ethnicity of all patients was White British.

ID	Age at baseline [years]	Breast cancer type	Receptor status			Nuclear grade	Breast density	Time between baseline ^{13}C-MRI and start of treatment [days]	Time between start of treatment and second ^{13}C-MRI [days]	Time between start of treatment and surgery [days]	Treatment schedule	Pathological response at surgery
			ER	PR	HER2							
1	63	IC NST	- (0/8)	- (0/8)	- (1+)	3	ACR B	11	7	155	Paclitaxel (weekly) x 12 with Carboplatin (three-weekly) x 4 cycles, then Epirubicin/ Cyclophosphamide (three-weekly) x 3 cycles; plus PARP inhibitor	non-pCR
2	56	IC NST	+ (8/8)	+ (4/8)	+ (2+; FISH positive)	3	ACR B	36	7	165	Docetaxel (three-weekly) x 4 cycles (dose reduction for cycle 4) followed by Epirubicin/ Cyclophosphamide (three-weekly) x3 cycles, alongside Trastuzumab and Pertuzumab (three-weekly).	non-pCR
3	32	IC NST	- (0/8)	- (3/8)	- (0)	3	ACR D	2	7	132	Paclitaxel (weekly) x12 with Carboplatin (three-weekly) x 4 cycles, then Epirubicin/ Cyclophosphamide (three-weekly) x 2 cycles; 20% dose reduction in Paclitaxel for cycle 4 and in EC for cycle 2 and one cycle omitted due to side effects.	pCR
4	49	IC NST	- (3/8)	- (0/8)	- (0)	3	ACR B	12	7	161	Paclitaxel (weekly) x12 with Carboplatin (three-weekly) x4, then Epirubicin/Cyclophosphamide (three-weekly) x3	non-pCR
5	50	apocrine IC	- (0/8)	- (0/8)	+ (2+; FISH positive)	3	ACR C	4	8	118	Docetaxel (three-weekly) x 4 cycles (dose reduction for cycle 4) followed by Epirubicin/ Cyclophosphamide (three-weekly) x 1 cycle (two more cycles omitted due to COVID-19 pandemic), alongside Trastuzumab and Pertuzumab (three-weekly).	pCR
6	43	IC NST	- (0/8)	- (0/8)	- (0)	3	ACR B	1	9	130	Paclitaxel (weekly) x12 with Carboplatin (three-weekly) x4, then Epirubicin/ Cyclophosphamide (three-weekly) x2 (one more cycle omitted due to COVID-19 pandemic); plus PARP inhibitor	pCR
7	54	IC NST	+ (8/8)	+ (8/8)	+ (3+)	3	ACR B	3	11	145	Docetaxel (three-weekly) x 6 cycles alongside Trastuzumab/Pertuzumab; no switch to EC due to adverse effects.	non-pCR

Supplementary Table 2. Results of ^{1}H and ^{13}C-MRI for all seven patients at baseline and follow-up. NA = not available. k_{PL} could not be calculated in one patient due to a technical failure. In another patient, failed fat saturation during the DCE-MRI did not allow reliable pharmacokinetic modelling.

ID	Timepoint	Volume [mL]	SNR_{PYR}	SNR_{LAC}	LAC/PYR	k_{PL}	K^{trans} [min^{-1}]	k_{ep} [min^{-1}]	v_e	$iAUC_{90}$	D [mm^2/s]	f
1	baseline	1.86	38.8	0.9	0.053	0.0126	0.346	0.855	0.410	0.331	0.92	0.18
	follow-up	1.85	60.6	5.2	0.111	0.0218	0.467	0.874	0.549	0.562	1.16	0.18
2	baseline	3.22	5.7	8.4	0.478	0.0015	0.193	0.497	0.442	0.269	0.46	0.18
	follow-up	2.12	2.3	2.6	0.400	0.0009	0.163	0.296	0.578	0.235	0.65	0.13
3	baseline	2.81	2.9	1.6	0.338	-	0.145	0.576	0.288	0.166	0.59	0.11
	follow-up	1.79	2.4	1.6	0.407	-	0.144	0.532	0.298	0.174	0.97	0.07
4	baseline	1.89	7.4	6.4	0.379	0.0035	0.352	1.072	0.322	0.338	0.58	0.15
	follow-up	1.44	3.6	4.2	0.420	0.0029	0.383	0.991	0.409	0.404	0.88	0.20
5	baseline	10.02	55.0	8.3	0.060	0.0032	0.505	1.064	0.486	0.513	0.95	0.13
	follow-up	5.66	19.2	4.8	0.162	0.0028	0.453	0.524	0.865	0.622	1.19	0.12
6	baseline	3.21	8.1	5.4	0.259	0.0024	-	-	-	-	0.63	0.12
	follow-up	1.84	0.5	1.9	0.665	0.0077	-	-	-	-	0.75	0.16
7	baseline	8.86	20.0	17.8	0.389	0.0148	0.308	0.727	0.423	0.373	0.57	0.22
	follow-up	4.71	27.0	11.3	0.230	0.0110	0.211	0.720	0.355	0.193	0.62	0.17

Supplementary Table 3. Correlation of ^{13}C-MRI and ^{1}H-MRI parameters where only cases with identical acquisition of ^{13}C-MRI data were included. Significant correlations are printed in bold.

	volume		K^{trans}		k_{ep}		v_e		$iAUC_{90}$		D		*f*	
	**r**	**P**	**r**	**P**	**r**	**P**	**r**	**P**	**r**	**P**	**r**	**P**	**r**	**P**
**Summed SNR_{PYR}**	0.87	**0.001**	0.54	0.135	0.41	0.272	0.03	0.937	0.38	0.311	0.37	0.290	-0.16	0.654
**Summed SNR_{LAC}**	0.68	**0.030**	-0.07	0.852	0.17	0.664	-0.37	0.329	-0.13	0.731	-0.35	0.325	0.61	0.065
**LAC/PYR**	-0.63	**0.049**	-0.50	0.169	-0.26	0.496	-0.26	0.494	-0.44	0.236	-0.48	0.163	0.44	0.199
**k_{PL}**	0.42	0.231	-0.12	0.768	0.11	0.785	-0.33	0.392	-0.19	0.625	-0.24	0.498	**0.65**	**0.044**

Supplementary Table 4. Correlation of percentage change of ^{13}C-MRI and ^{1}H-MRI parameters. The significant correlation is printed in bold.

**%change of**	**LAC/PYR**		k_{PL}		**volume**		K^{trans}		k_{ep}		v_e		$iAUC_{90}$		**D**		*f*	
	**r**	**P**	**r**	**P**	**r**	**P**	**r**	**P**	**r**	**P**	**r**	**P**	**r**	**P**	**r**	**P**	**r**	**P**
**LAC/PYR**			0.63	0.181	0.09	0.853	0.47	0.351	-0.40	0.437	0.84	**0.035**	0.72	0.106	-0.31	0.495	0.42	0.352
**k_{PL}**	0.63	0.181			0.05	0.925	0.86	0.061	0.50	0.393	0.14	0.819	0.83	0.080	-0.34	0.510	0.56	0.248

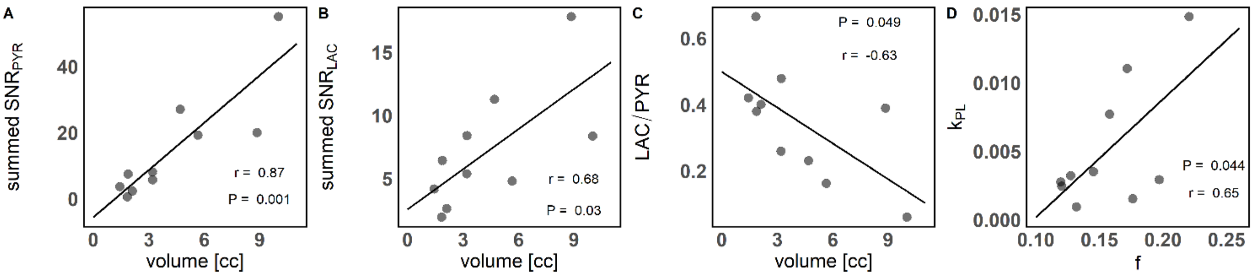

Supplementary Figure 1. Correlations of ^{13}C-MRI parameters with volume and perfusion fraction *f*.

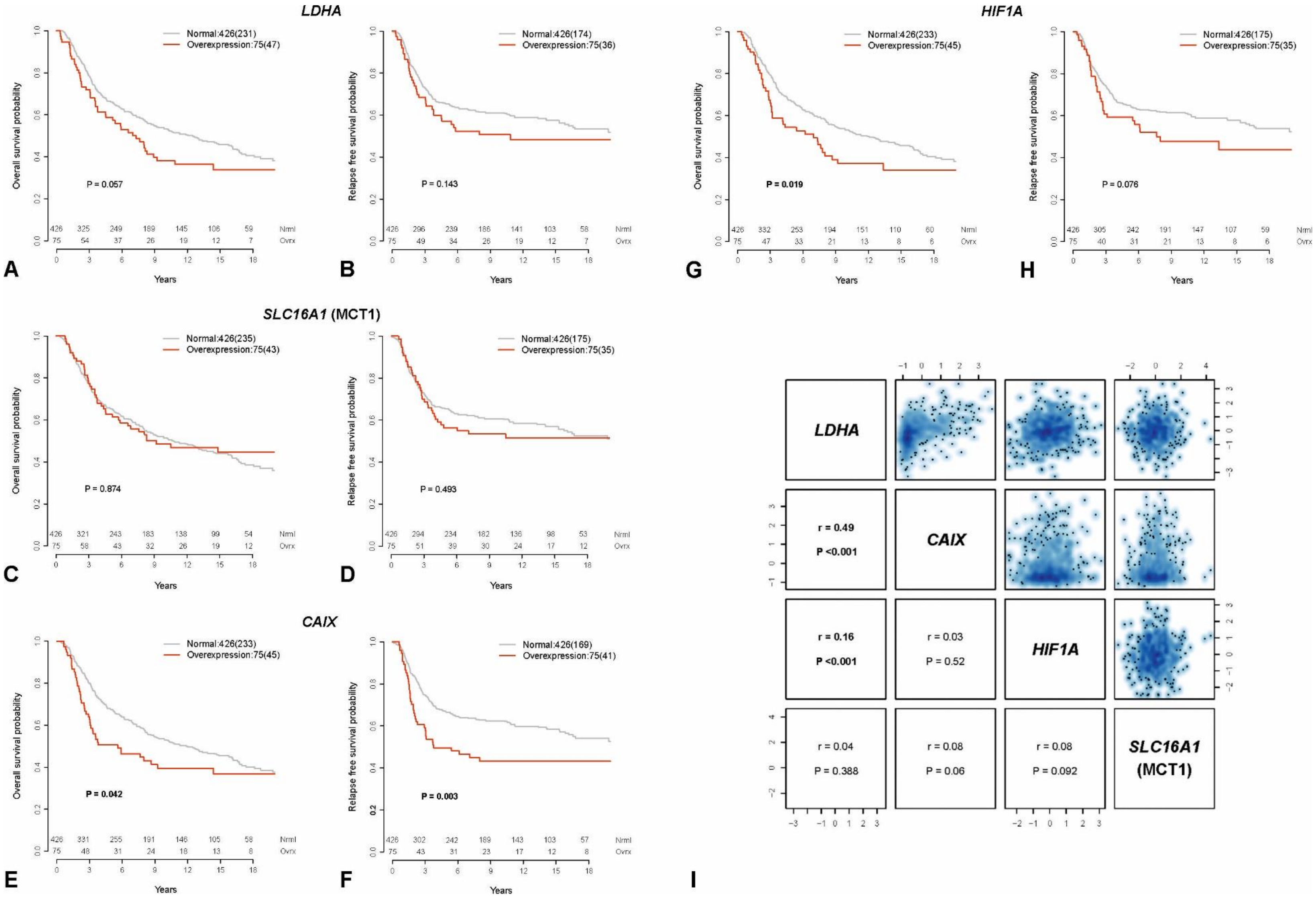

Supplementary Figure 2. Correlation of *LDHA*, *SLC16A1* (MCT1), *CAIX*, and *HIF1A* expression with survival (A-H) and the correlation matrix of *LDHA*, *SLC16A1* (MCT1), *CAIX*, and *HIF1A* expression (I) in a subset of the METABRIC cohort including the tumor subtypes represented in this study. Kaplan-Meier curves for normal expression and overexpression (85th percentile) of *LDHA* (A and B), *SLC16A1* (MCT1) (C and D), *HIF1A* (E and F), and *CAIX* (G and H) are shown. The left column shows overall survival and the right column relapse free survival. Number of events are shown in bracket. There is a significant correlation between LDHA and MCT expression (z-scores) with the hypoxia markers CAIX and HIF1A (I). r: Pearson correlation coefficient.

References

1. Bedair R, Graves MJ, Patterson AJ, McLean MA, Manavaki R, Wallace T, et al. Effect of Radiofrequency Transmit Field Correction on Quantitative Dynamic Contrast-enhanced MR Imaging of the Breast at 3.0 T. Radiology. 2015;279:368–377.
2. Sacolick LI, Wiesinger F, Hancu I, Vogel MW. B1 mapping by Bloch-Siegert shift. Magn Reson Med. 2010;63:1315–22.
3. Fedorov A, Beichel R, Kalpathy-Cramer J, Finet J, Fillion-Robin J-C, Pujol S, et al. 3D Slicer as an image computing platform for the Quantitative Imaging Network. Magn Reson Imaging. 2012;30:1323–41.
4. Fritz-Hansen T, Rostrup E, Larsson HBW, Søndergaard L, Ring P, Henriksen O. Measurement of the arterial concentration of Gd-DTPA using MRI: A step toward quantitative perfusion imaging. Magn Reson Med. John Wiley and Sons Inc; 1996;36:225–31.
5. Tofts PS. Modeling tracer kinetics in dynamic Gd-DTPA MR imaging. J Magn Reson Imaging. 1997;7:91–101.
6. Bedair R, Priest AN, Patterson AJ, McLean MA, Graves MJ, Manavaki R, et al. Assessment of early treatment response to neoadjuvant chemotherapy in breast cancer using non-mono-exponential diffusion models: a feasibility study comparing the baseline and mid-treatment MRI examinations. Eur Radiol. 2017;27:2726–2736.
7. Grist JT, McLean MA, Riemer F, Schulte RF, Deen SS, Zaccagna F, et al. Quantifying normal human brain metabolism using hyperpolarized [1–13C]pyruvate and magnetic resonance imaging. Neuroimage. 2019;189:171–9.
8. Gallagher FA, Woitek R, McLean MA, Gill AB, Manzano Garcia R, Provenzano E, et al. Imaging breast cancer using hyperpolarized carbon-13 MRI. Proc Natl Acad Sci. 2020;117:2092–2098.
9. Schneider CA, Rasband WS, Eliceiri KW. NIH Image to ImageJ: 25 years of image analysis. Nat Methods. Nature Publishing Group; 2012;9:671–5.
10. Patro R, Duggal G, Love MI, Irizarry RA, Kingsford C. Salmon provides fast and bias-aware quantification of transcript expression. Nat Methods. 2017;14:417–9.
11. Robinson MD, McCarthy DJ, Smyth GK. edgeR: a Bioconductor package for differential expression analysis of digital gene expression data. Bioinformatics. 2010;26:139–40.
12. Ritchie ME, Phipson B, Wu D, Hu Y, Law CW, Shi W, et al. limma powers differential expression analyses for RNA-sequencing and microarray studies. Nucleic Acids Res. 2015;43:e47.
13. Leek JT. svaseq: removing batch effects and other unwanted noise from sequencing data. Nucleic Acids Res. 2014;42:e161.
14. Therneau TM, Grambsch PM. Modeling Survival Data: Extending the Cox Model. New York SSMN, editor. Springer Science+Business Media New York; 2000.
15. Harrell FEJ. Regression Modeling Strategies. Springer Science+Business Media New York; 2001.
16. Reston VA. The American College of Radiology (ACR) (2013) Breast Imaging Reporting and Data System Atlas (BI-RADS® Atlas). 2013.
